# Understanding Antecedents of Nurses' and Physicians' Workaround Behavior Regarding Hospital Information Systems: Qualitative Interview Study

**DOI:** 10.2196/51781

**Published:** 2025-07-15

**Authors:** Eileen Doctor, Jasmin Hennrich, Torsten Eymann, Christoph Buck

**Affiliations:** 1 Branch Business and Information Systems Engineering of the Fraunhofer FIT, Wittelsbacherring 10, Bayreuth, 95444, Germany; 2 FIM Research Center for Information Management, University of Bayreuth, Bayreuth, Germany; 3 University of Applied Sciences Augsburg, Augsburg, Germany

**Keywords:** workaround, hospital information system, grounded theory, qualitative interview, time-critical routine, hospital, ward, accuracy, innovation, antecedent, patient safety, nurse, physician, behavior, health information, medical personnel

## Abstract

**Background:**

Hospital information systems (HISs) aim to support users in their time-critical routines on hospital wards with accurate and timely information. However, if these systems create blockages to workflows, nurses and physicians develop workarounds to provide care to the patients, nonetheless. Workarounds are considered negatively when associated with risks and positively when seen as feedback and a source of innovation. Learning about the antecedents of workarounds allows for the establishment of control mechanisms, under the promise of enhanced patient safety.

**Objective:**

This study seeks to explore which antecedents shape nurses’ and physicians’ workaround behavior in the context of HISs, how they influence behavior and interrelate, and the intentions with which they are carried out.

**Methods:**

Using 26 qualitative interviews with nurses, physicians, and health information technicians from Germany and the United States and applying grounded theory analysis techniques, we identify antecedents of HIS-related workarounds and respective relations.

**Results:**

From the interview transcripts, we derive 506 open codes which we cluster into 3 direct causes (organizational prerequisites, human factor, and system), and 4 influencing factors (regulations, sector funding, role of software providers, and role of ownership and management). While Influencing Factors constitute higher-level influences, they do not directly impact nurses' and physicians' behavior but rather depict the defaults that lead to conditions for Direct Causes of workarounds.

**Conclusions:**

This study provides an understanding of the antecedents of workarounds performed by medical personnel regarding HIS use, structures and categorizes them, and lays the foundation for an understanding of users’ deviant behavior. Moreover, by revealing cause-effect relationships between the antecedents, we take on a behavioral perspective and provide a basis for developing effective strategies to prevent the need for workarounds. We contribute to the research stream of workarounds in health care and emphasize that once the reported and derived direct causes and influencing factors of workarounds have been tackled, working conditions, patient safety, and the overall quality of health care may improve under full digital support.

## Introduction

### Overview

Hospital information systems (HISs) promise to support their medical users in daily business through the collection, processing, and dissemination of medical and administrative data [1]. Common key features facilitating core and support processes include data provision (eg, administrative documentation, medical documentation, and reporting), results management (eg, laboratory and radiology reports), and electronic order transmission via computerized physician order entry [2]. HISs themselves depict sociotechnical systems, and therefore, their use depends not only on the technology, as the participants and organizational environment are critical to successful use [3].

**Figure 1.**
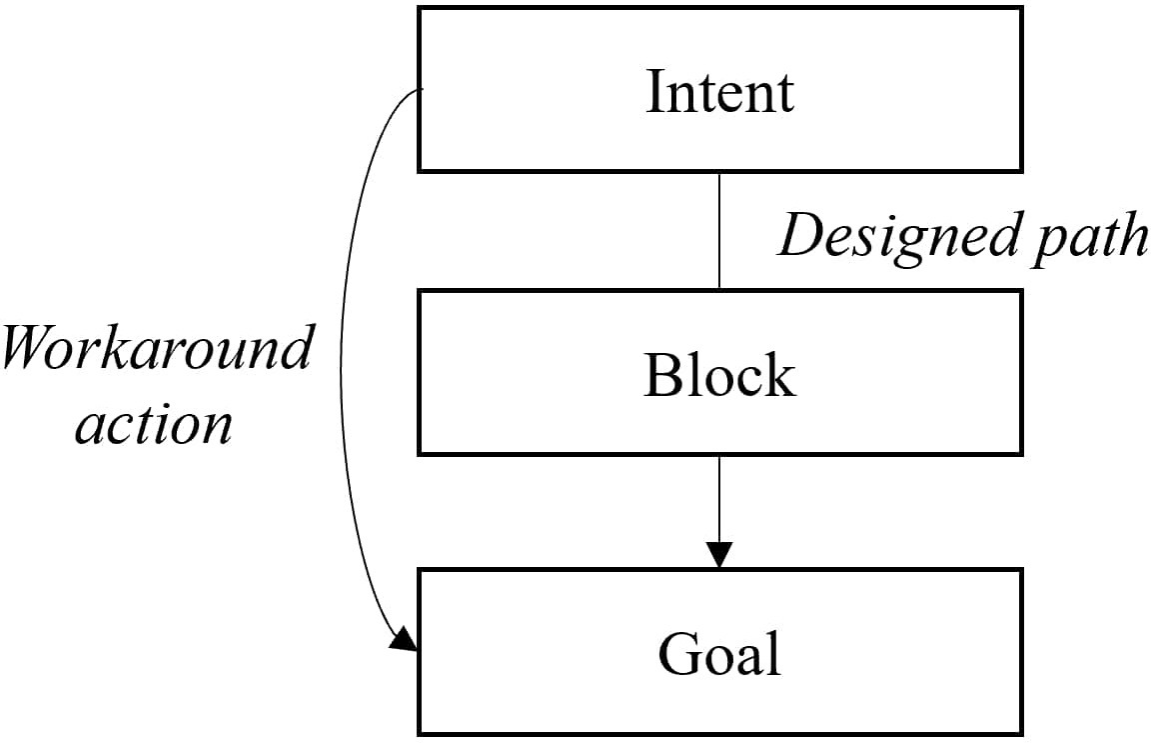
Workaround definition following Ejnefjäll et al [6].

Even though HISs promise to support the medical staff, in reality, it is often otherwise, and they are rather perceived as a hindrance than a support. Among others, a nonintuitive system design and slow information technology infrastructure can seriously impair nurses and physicians in their activities [4]. When HISs are perceived as obstructive, they do not depict nurses’ and physicians’ workflows, forcing them to engage in coping behavior—so-called workarounds [3]. Alter [5] describes situational constraints, obstacles, and anomalies as factors that nurture the perceived need for a workaround. Generally, in Alter’s [5] theory of workarounds, sequential steps lead to the emergence of workarounds. Their development represents a typical problem-solving process that initially starts with the identification of the need for a workaround, followed by the collection of potential workarounds, and then the selection thereof. After that, the workaround must be created and executed, eventually leading to consequences. Ejnefjäll et al [6] describe how researchers use the term commonly and distance the phenomenon from errors, mistakes, deviations, or shortcuts as a temporal view: “When the designed path is blocked, a workaround provides an alternative path to the same goal without completely removing the block” (Figure 1).

From a behavioral perspective, a workaround depicts the response to a perceived or real problem that an individual wants to either fix or avoid [7]. This off-track behavior is widespread, often consisting of a set of work practices not formally described in process models, rules, and regulations, such as omitting or adding steps and performing unauthorized actions to still reach a certain goal. In hospitals, this behavior may involve process shortcuts, such as circumventing medical documentation tasks, overruling role definitions and permissions through account sharing, potential data transfer outside of the HIS, and the illegitimate use of personal devices, to name a few [8]. Whenever workarounds occur, they serve as indicators for a mismatch between the involved information systems, defined processes, and user requirements [9]. Workarounds are considered to be ambiguous. Positively, they are seen as a source of feedback with an innovative character [10,11]; negatively, they are considered to be both noncompliant and high-risk [12]. In hospital care, workarounds can create a threat to the health and safety of patients [13] and occur in various forms. A commonly reported phenomenon, for example, is the circumvention of the system for efficiency and ease. Communicating important information via telephone instead of HIS is often rooted in a mismatch in system design and work routines and can lead to medication errors, mix-ups of patient records, effort in reworking, or privacy issues [14,15]. A lack of interoperability and standardization between systems further encourages verbal communication [16]. Patient information may be shared on paper or via private phone, if data sharing in the HIS is not possible [15] or if the workers simply do not possess the knowledge to do so [17].

The potential adverse effects of workaround behavior in patient care underscore the criticality of understanding the antecedents of their occurrence. This understanding is crucial in developing effective strategies to prevent the need for workaround behavior and promote safe and efficient health care practices.

### Workaround Behavior

To better understand the occurrence of workarounds, research has provided various theoretical lenses for workaround analysis, each including different definitions and extents of behavior [18-24]. The theory of planned behavior (TPB) is one of the prominent theories to explain behavior [18] and also lays the foundation for Alters’ theory of workarounds [5]. TPB allows for the concentration on explaining why an intention to engage in a certain human behavior is formed in a specific context. Workarounds depict a behavior that an individual performs to achieve a goal or task when the normal course of action is unavailable or unsuccessful. Therefore, this theory is prone to investigate the occurrence of workarounds, as demonstrated by Soffer et al [7], who apply the TPB to explain workaround decisions in the business context.

**Figure 2.**
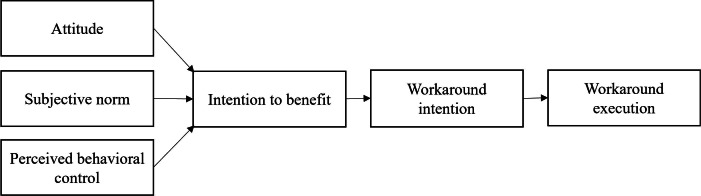
Theory of planned behavior combining Ajzen [18] and Soffer et al [7].

The TPB views the intention to perform a behavior as the direct antecedent of it [18]. According to TPB, the behavioral intention (in our example, the intention to perform a workaround) is determined by the attitude towards the behavior, the subjective norms, and the perceived behavioral control (Figure 2). The attitude refers to evaluating favor or disfavor against a behavior, which has expected outcomes, subjective risks, and benefits. The subjective norm refers to what others think of the behavior, whether most people approve or disapprove. Perceived behavioral control is the perception of ease or difficulty, the extent to which individuals believe they can control their own behavior.

By establishing a theoretical foundation rooted in the TPB, we not only direct our attention towards unraveling the intricate web of factors that influence workaround behavior within HIS but also strengthen our research endeavor with a time-tested framework renowned for its efficacy in explaining human behavior, from technology adoption to eating behavior, to name a few [25-27]. By studying the factors that influence workaround behavior upon a strong theoretical foundation, we create a basis to examine the interplay of cognitive, social, and contextual elements in the context of workarounds. Through this alignment of theoretical underpinning and qualitative investigation, we aim to understand how attitudes, subjective norms, perceived behavioral control, and other psychological constructs operate in tandem to shape individual behavior within the complex realm of HIS. This approach not only provides scholarly rigor but also generates insights into the enhanced functionality, efficiency, and integrity of HIS, thereby benefiting health care informatics, organizational management, and medical practitioners alike.

Further, we include Soffer et al’s [7] notion that a workaround is performed with the intention to benefit someone. Following their claim for contextual adaptations, we define the intention to benefit in the medical inpatient setting as either beneficial for the individuals performing the workaround themselves, as beneficial for the patients, the local unit (ward), or the organization. By integrating the intention to benefit, we extend the understanding of the underlying motivations for workaround behavior in relation to HIS in the inpatient sector.

Existing research on workarounds has already identified antecedents for workarounds; however, they are related to single modules of HIS, such as electronic health records [13,17], computerized physician order entry [28,29], computerized consultation [30], or medication administration systems [31]. Existing research provides observations and analyses of workarounds, underlying rationales, and possible solutions. Thus, it offers attributes and categorizations through a static lens [17,32], while this research article aims to examine the interplay and cause-effect relationships of potential influencing factors, leading to a more profound comprehension of workaround behavior, motivation, and underlying intentions. This approach, along with qualitative insights, enables us to grasp the psychological and social dynamics that shape health care professionals’ behaviors within the human-centered context of HIS.

### Goal of This Study

The purpose of this study is to examine how underlying antecedents shape individual workaround behavior in the context of HIS. By focusing on the root causes of workaround behavior, we integrate cognitive, social, and contextual elements to highlight the complex intentions behind these actions. This approach allows us to delve deeper into factors like system design flaws, organizational constraints, and user dissatisfaction that drive workaround behavior. Unrevealing this knowledge can bring attention to working conditions, facilitate HIS and workflow redesign, and push the development of more fitting guidelines.

## Methods

### Data Collection

To identify the antecedents of workarounds related to HIS, we conducted semistructured interviews. With this research strategy, the objective is to obtain in-depth, real-world depictions from frontline users of HIS and feature detailed and deep information [33,34]. Thereby, we adhere to the interpretative paradigm and use qualitative methods to understand nurses’ and physicians’ behavior, emphasizing the complexity of process design and personal motivation [35]. Semistructured interviews are guided with an explicit structure through which the participants can access their experiences and reflect upon the underlying antecedents in a layered introspective journey [36].

The data collection process involved a series of ongoing and repeated steps that worked together harmoniously. After deriving thematic clusters from the research objective, we discussed the content of an interview guideline with the author team. The final version of the interview guideline is structured in 5 clusters. The first section includes the mutual introduction of the interviewer and the presentation of the research idea. This is followed by the second cluster, covering questions regarding the participant’s age, job, and daily routine. The third cluster bundles questions about the individual’s intersections with HIS in everyday activities, their confidence in handling HIS, and the interviewees’ perception of the fit of the HIS towards their needs. Questions in cluster 4 gently approach the topic of workarounds, asking indirectly about the way the interviewees handle HIS misfit, as well as the rationales they have for their deviating behavior. In the last cluster, the interviewees had the chance to add input that was not explicitly addressed. Before the first interview, we pretested the guideline with a health economist, a business information systems researcher, a nurse, and a physician. We evaluated the guide after each interview and iteratively developed it further. Any misconceptions were cleared up and integrated into 4 iterations of the interview guide, which led to more focused questions about the research topic and better information for the participants during the interview. Therefore, we followed the recommendations of Corbin and Strauss [37]. Further, we selected and recruited participants via convenience sampling. For recruitment, we used convenience sampling and contacted hospitals where we had established relationships through previous work, such as internships, projects, and personal contacts. We then followed a snowball sampling approach to expand our participant pool. To ensure depth of information, we relied on nurse coordinators and chief physicians who recommended medical practitioners actively involved in HIS implementation projects. It is important to note that we did not involve organizational functions such as workers’ councils in the recruitment process. Our focus was on individuals with direct experience with HIS, and we believed that engaging workers’ councils could have introduced unnecessary procedural complexities and potential biases. Instead, we prioritized recruiting participants through trusted clinical leaders who were intimately familiar with their teams’ work practices and could identify individuals deeply engaged with HIS. In recruitment, we determined the eligibility criteria to include participants who are frontline health care professionals, specifically nurses and physicians, with regular use of HIS and experience with workaround behaviors. A diverse range of ages, genders, work experience, and HIS proficiency was included to ensure varied perspectives on system use and workarounds. Further, we paid attention to a representation of age groups, gender distribution, levels of work experience, and different HIS capabilities (determined by a guided self-estimation). We contacted individuals directly, as well as with an approach of contacting C-level management with email requests that provided a short description of the research purpose and further relied on personal network contacts. Additionally, we asked participants about colleagues who could be interested in participation. To optimize interview time, we provided participants with a detailed introduction via email or telephone beforehand, allowing the interviews to focus on gathering substantive information. As we were aware of participants’ potential fears of disciplinary consequences when truthfully explaining their workaround behavior, we included the recommendations and strategies of Bergen and Labonté [38] to detect and limit social desirability bias and strongly distanced ourselves from management as neutral researchers. The interviews took place both via telephone and face-to-face. We preferred personal contact over audio contact to create a trusting atmosphere and extract more detailed information [39]. After consent was obtained from the participants, an audio recording of the interviews was made, the transcription of which enabled a thorough data analysis. Transcribing and coding the data led to more reflection and revision of the process as needed. The software MAXQDA 2020 (Verbi), which was specially developed for qualitative and mixed methods of data analysis, was used for this purpose. Since this was an iterative process, it was not strictly linear or in a clear timeline. For example, we started the analysis of the transcripts while still conducting interviews [40]. The constant comparison and back-and-forth nature of the process aimed to build a thorough and ever-expanding understanding of the participants’ experiences and perceptions, even if these feelings were not always easily observable or distinct. We concluded the iterative data collection process upon reaching data saturation, following Nelson’s [41] “conceptual depth,” which has already proven itself in the research of behavioral factors [42]. At this point, additional data collection yielded no new themes or insights, indicating that we had achieved a comprehensive understanding of the research topic and further coding was no longer feasible, ensuring that our findings were both robust and exhaustive.

### Data Analysis

We performed the data analysis by applying grounded theory analysis techniques. By using the grounded theory methodology (GTM) approach, researchers can effectively manage unstructured qualitative datasets. They are able to identify relevant categories and relationships within the data while providing meaningful context and interpretation [40]. As per GTM, the analysis starts with the initial set of collected data. The findings from the initial interview impact the researcher, consequently shaping the approach taken in the subsequent interview sessions. Throughout the interview procedure, the participants’ answers were carefully reviewed and verified with cross-references [43]. Any misunderstandings during this went into the refinement of the interview guidelines. Moreover, this approach facilitated the clarification and precise alignment of the research question [40].

While using the GTM and adhering to the 3-step Straussian approach encompassing open, axial, and selective coding, we began by breaking the qualitative datasets down into relevant fragments (open coding). After an initial read of the transcripts, we highlighted phrases that were relevant to the research topic, resulting in 506 open codes. Following GTM techniques [44], we examined the codes and merged common topics into concepts. After ensuring an even allocation and hierarchy of the concepts, we merged them into categories and identified relationships (axial coding). Thereby, we distinguished the core category antecedents to HIS-related workarounds from other categories (selective coding) [40]. In order to validate the coding results, 2 authors performed a card-sorting allocation. The first author identified open codes and concepts, which served as the basis for the second author to conduct a blind card-sorting round. The second author added not initially identified open codes along the process. Whenever there were discrepancies in the allocations of the authors, the authors discussed the results to find agreements. The coding process was iteratively refined through constant comparison and adjustments, and the open coding steps were repeated backward and forward whenever new insights were gained [40].

The coding process is condensed into a coding catalog, which serves to enhance transparency and facilitate understanding for fellow researchers. In this catalog, each code is accompanied by its name, definition, an example, and rules for its appropriate application. This compilation empowers other researchers to code supplementary interviews using the catalog as a guide. An example is provided in Multimedia Appendix 1.

The study is reported following the applicable study design and reporting guidance, the Standards for Reporting Qualitative Research [45] (Checklist 1).

### Ethical Considerations

Given the nature of the research, which centered on the professional practices of nurses and physicians without intervention or impact on patient care or professional work practices, and after ethics advisory consultation prior to the research based on the internal guideline “Forschen in gesellschaftlicher Verantwortung” (FIVER), an external ethics approval process was not deemed necessary according to the institutional practices of the Fraunhofer FIT.

All participants were informed about the research objectives, procedures, and their impact before the interviews began. Participants provided informed consent in written form, agreeing to share their experiences. Participants were made aware of their right to opt out of the study at any time without any consequences, and their anonymity was strictly maintained throughout the process. No secondary data or pre-existing datasets were used in this study, so additional consent for secondary analysis was not required.

The collected data were anonymized to the full extent, and no compensation was provided for the voluntary participation in the study.

## Results

### Descriptive Results and Study Population

In total, we conducted 26 interviews in 3 hospitals in Germany and 1 in the United States with a mean duration of 29 minutes. The interviews were conducted primarily in off-peak times, such as before or after shifts or during lunchtime. The study sample consisted of 26 participants, including 14 (53.85%) physicians, and 9 (34.62%) nurses, and we were able to enrich the study set with 3 (11.54%) health information technicians (HIT). The participants had a mean age of 44.1 years, with an average of 20.3 years of job experience. The sample was slightly female-dominated, with 15 (57.7%) of the participants being women. We were able to recruit HIT employees in the US American setting, as the participating hospital served as a pilot hospital for the HIS software provider and created this role specifically for the development of the HIS. In comparison, the participating German hospitals assigned the task of HIS support to the regular IT department, without explicit consideration in workforce planning. The average age varied across roles: HITs had the lowest average age at 34.3 years, followed by nurses at 43.4 years, and physicians at 46.6 years. All participants were experienced and had at least 2 years of work experience within the field. Participants younger than the age of 30 years were more evenly distributed among the roles, with 2 (40%) being physicians, 2 (40%) nurses, and 1 (20%) HITs. In the 30‐50 years age group, the majority were physicians (n=6, 54.5%), followed by nurses (n=3, 27.3%) and HITs (n=2, 18.2%). Among participants older than 50 years, the majority were also physicians (n=6, 60%), with the remaining (n=4, 40%) being nurses. A total of 6 (23%) participants rated their HIS confidence as moderate (meaning they can use the HIS in dictated procedural flows and, above that, rely on the help of colleagues), 13 (50%) participants rated as solid (meaning they trust their skills, use the system easily and feel confident in helping others), and 7 (27%) participants rated as expert (meaning they operate HIS easily, engage in HIS redesign, create their own filters and templates, and initiate system changes), out of which 3 participants were HITs. The descriptives are reported in Table 1.

**Table 1.** Sample characteristics.

Characteristics	Details
Number of interviews	26
Mean duration (mins)	29
Sample composition, n (%)	
Physicians	14 (53.85)
Nurses	9 (34.62)
HITs^a^	3 (11.54)
Mean age (years)	44.1
Mean job experience (years)	20.3
Role distribution by age group, n (%)	
Below 30 years	
Physicians	2 (40)
Nurses	2 (40)
HITs	1 (20)
30‐50 years	
Physicians	6 (54.5)
Nurses	3 (27.3)
HITs	2 (18.2)
Older than 50 years	
Physicians	6 (60)
Nurses	4 (40)
HIS^b^ confidence levels, n (%)	
Moderate	6 (23)
Solid	13 (50)
Expert	7 (27)

a HIT: health information technician.

b HIS: hospital information system.

### Three-Step Coding Results

**Figure 3.**
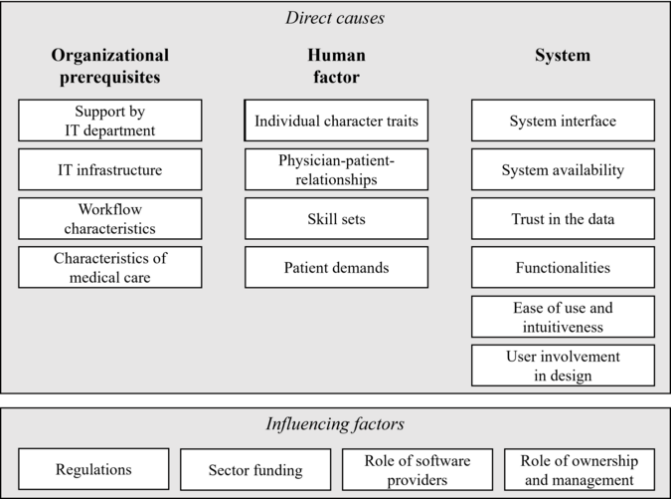
Overview of categories and concepts derived from the interviews.

In the following, we describe the 18 antecedents of HIS-related workarounds, as derived from our qualitative data sets. The following overview shows all categories and concepts from the interview study (Figure 3), which is followed by a detailed description of the antecedents of workarounds, supported by interview quotes and exemplary workarounds, whenever applicable.

### Organizational Prerequisites

The organizational prerequisites category consists of 4 concepts: support by IT department*,* IT-infrastructure*,* workflow characteristics, and characteristics of medical care.

#### Support by IT Department

Interviewees perceived the support and availability of the IT department as determining their workaround behavior. This is, for example, because the organization may not synchronize the shift schedules of medical staff and the working hours of IT staff. However, this lack of organizational prerequisites can lead to workarounds since problems with IT do not only occur during office hours but also at night or on weekends. In particualr, in the nighttime, when there is low IT support and a low practitioner ratio, participants reported high improvisation of, for example, using paper-based notes and transferring these memos into the system after the issue is solved, often after their shift. They report a lack of support as:

*Yes, at night it becomes difficult, there is an emergency on-call service, but it is only for emergencies.*

[interview 2, position 15, open code: availability/ expertise of IT support and staff]

#### IT Infrastructure

Participants mentioned the overall level of IT infrastructure as vital for their performance and as a reason to deviate from existing processes. For example, as stated in the open code “insufficient number of workstations,” participants attribute a perceived loss of time to the lack of workstations and the need to share them among all in the respective shift, leading to workarounds to avoid waiting times (interview 16, position 125‐130). These workarounds include, for example, sharing of login data to reduce the login and logout processes. Furthermore, the IT equipment is often not only insufficient in quantity but also very outdated:

*I am the first to arrive [...] in the morning, which means that I come to the ward in the morning and the PCs are not switched on, then I have to start the computers, which takes three to eight minutes.*

[interview 5, position 33, open code: login authorization]

#### Workflow Characteristics

Interviewees perceive routines being designed in a way that does not support them in their HIS documentation tasks during their workflows. For example, it is the situation that the same data has to be entered more than once into the HIS, sometimes also with the need to reevaluate colleagues’ input, causing inefficiencies (interview 12, position 45, open code: validation of each department’s own effort [evidence]). Still, because of time pressure and the participants questioning the sense of purpose of these workflows, they deviate and show the workaround of dropping documentation tasks under the promise that someone else will catch the missing data:

*It’s true that you deliberately omit this if you know your patient will be admitted as an inpatient anyway. If so, I often leave out the status or don’t write a detailed anamnesis in the system; […] because I know that everything will be thoroughly documented again.*

[interview 12, position 47, open code: time-saving and efficiency]

#### Characteristics of Medical Care

Participants describe medicine to be highly complex and personalized, as “unpredictable imponderables, [with the] need for quick adjustment” (open code), and thus, in need of flexibility to meet quickly emerging needs. Consequently, the needs require spontaneous and immediate responses by providers, a relationship of a subjugating nature. Users engage in workarounds when they have to correspond to these emerging needs, and existing HIS hinder their workflows, for example, with the workaround of using data entry fields by nurses for the communication of urgent patient information, which were not intended for this purpose (interview 3, position 149, open code: time-saving and efficiency).

### Human Factors

We divide this category into 4 concepts as follows: individual character traits, skillset, physician-patient relationship, and patient demands*.*

#### Individual Character Traits

Age, fears, affinity to technology, individual preferences, interpersonal relations and responsibilities, and habits are further reasons for workarounds. These individual aspects commonly favor paper-based workarounds, evident in manual documentation that needs to find its way back into the system. The participants highlighted:

*Okay, of course, I am from the old generation, which is not so fit on the computer, [the use of HIS] always takes me a bit longer.*

[interview 7, position 46, open code: age and affinity of technology]

Besides age and affinity to technology, individual preferences, habits, and insecurities of the medical staff are another reason for the occurrence of workarounds:

O*ne is the way I have always done it right. I want to try to do it the way I was taught*

[interview 25, position 70, open code: habit/ experience/improvisation]

#### Skillset

Respondents stated they are left without support when using newly introduced systems, which means they often do not have the right skill set to work with the HIS and use all its functions. In these situations, participants responded that they often rely on workarounds, such as handing over tasks to colleagues and asking colleagues for help, even omitting necessary steps, to account for the (needed) skillset:

*That’s another problem with introducing these [systems]. [...] When things are introduced here, there is no change manager to say, okay, what are the processes, and how do we introduce this. Instead, they say: 'We have the system now, please all work with it from today’.*

[interview 14, position 65, open code: no instruction or training-on-the-job]

There is hardly any support for the introduction of the system and regarding the familiarization with new functions of the HIS:

*[There are] few trainings, they always prefer the snowball system.*

[interview 9, position 20, open code: no instruction/ training-on-the-job]

#### Physician-Patient Relationship

Medical practitioners take responsibility for the overarching goal of providing the best possible care for patients. Respondents describe the nature of medicine and care as a reliable, personal, humanistic, and caring act, in which the physician-patient relationship is highly valued. However, when confronted with the time-consuming use of HIS, they fear a deterioration of this relationship, which they want to avoid. Hence, they describe workarounds that aim to reduce the time with the system, often leading to reworking and touch-ups after their shift:

*My honor and my desire to work in a meaningful, interpersonal, and humanistic way suggests that I try to reduce the time spent on the computer to the bare minimum.*

[interview 5, position 45, open code: value of doctor-patient-relationship]

Interviewee 3 confirmed:

*So for me, whenever it’s a really stressful day, treating my patients is my top priority. And I reduce documentation, or leave things out, […] because I am more concerned with making sure the people are taken care of.*

[interview 3, position 67, open code: prioritization]

#### Patient Demands

This concept describes how the demands of the patients can trigger workarounds. For example, the shift in patients’ self-perception can lead to differing demands on health services. Patients wish to be well informed about their medical history and treatment, which the HIS must be able to depict, and if this is not the case, the staff turns to, for example, paper-based workarounds:

*When the administration complains that we need so much paper and toner [for printing out], I then explain that we have to show the patient what we have done. 'But, why?' [they ask], 'because the patient of today wants to know exactly what he [or she] has’.*

[interview 4, position 257, open code: patient autonomy and demands]

### System

This category includes the following concepts: system interfaces, system availability, trust in data*,* functionalities, ease of use and intuitivity, and user involvement in design.

#### System Interfaces

Nurses’ and physicians’ workarounds can be caused by the lack of interfaces between the different systems used in the hospital. Organizationally reinforced HIS barriers can create cumbersome hurdles, which lead to workarounds such as printing out snapshots for other providers, screenshotting for manual data entry, and relying on other communication paths, such as calls. They report:

*The fact is that we will always have subsystems, but there is the problem of interfaces from both directions. […] If I change an element in one of the systems, then I want it to [match] since I already have to operate two systems.*

[interview 14, position 45, open code: no data exchange between subsystems]

This barrier can lead, for example, to status inquiries by phone or faxing of medical reports, which are then printed out and scanned again (interview 12, position 33, open code: mailing/faxing/calling external providers).

#### System Availability

The existence and accessibility of the HIS depict a prerequisite for its use. Users describe slow system responsiveness or regular system updates. In all reported cases, issues with system availability led to waiting and then paper-based interim documentation and communication with time-intensive reworking after the regular shift. There may also be repetitive cutbacks in HIS availability:

*[…] We get an email saying ’We have to install an update tomorrow, so […] you can’t take lab results, you can’t perform X-rays, […] you can’t discharge patients, you can’t do anything.*

[interview 6, position 67, open code: frequent updates impact routines]

When the respondents find themselves confronted with system unavailability, they work around the actual blockage in their workflow and rely on paper-based interim solutions, which require all information to be subsequently transferred to the HIS (interview 11, position 57, open code: paper-based interim solutions when system is down).

#### Trust in Data

The participants expressed their concerns about data correctness in the HIS. Users’ perceptions of HIS being a reliable source of information and useful support are reportedly influenced by the fluctuation of the frontend design and the actuality of data within the HIS. Any distrust toward data collection and storage can cause users to perform workarounds, for example, involving unnecessary repetitions of diagnostic procedures. This may happen, even if the diagnostic results are saved in the HIS already, just to ensure that the, for example, radiological images they rely on in their diagnoses are up to date. When data transfer is unreliable due to internet connectivity problems, for example, during rounds where the Wi-Fi connection is not ensured in all patient rooms, data quality issues occur in the system:

*I can’t rely on anything at all to be up to date.*

[interview 4, position 87, open code: no (trust in) data actuality]

#### Functionalities

The existence or absence of alarming functionalities was also mentioned as a source of workaround behavior, leading to the workarounds of calls instead of relying on the systems.

*If someone has a low HB value, the system knows this and marks it [in color] red, but there is no notification, and therefore, nobody is aware. […] That’s why the laboratory has the instruction to call the corresponding requester or to inform the ward. [The information] goes through ten hands, which is actually nonsense, that’s not what the system is for.*

[interview 4, position 85; open code: workaround - calling for urgent results]

Further, HIS has no functionality for collaborative work, such as shared editing of patient charts, leading to the workaround of custom-built lists and shadow IT:

*We built an Excel list and put all patients and rehab requests in this list and made columns for [...] [the individual steps of the workflow]. […] This is what [HIS] in its present version cannot provide.*

[interview 6, position 25, open code: mid-process transparency (live status)]

#### Ease of Use and Intuitivity

Under this category, we subsume statements dealing with aspects of data presentation and handling, for example, visual or task-related preferences of what individuals need on their screens. Respondents highlighted precision in HIS searching patterns and the preparation of search results. These include, for example, the necessity of remembering keywords of form headings and other specific search terms (interview 17, position 61‐63, open code: need for implicit knowledge to find search terms). Further, physicians need an overview of specific attributes of patients, collectively summarized for tracking purposes, instead of focusing on a single case reference:

*The biggest weakness of [HIS] is its enormous focus on individual patients. I can’t create lists about problems, about time frames, anything like that. But that would be really important […].*

[interview 6, position 53, open code: single case reference]

#### User Involvement in Design

The participants were concerned with the role that they and their needs partake in the design of HIS. This includes how users are systematically encouraged to help shape the system, as well as system designers’ ways of including individual feedback and design requests of users:

*Well, the systems are usually created by people who have never done the work themselves.*

[interview 12, position 37; open code: designed by nonmedical staff, thus misfit]

Moreover, they feel as though they support the HIS through their laborious entries instead of being supported by the HIS in their daily medical workflows, which further adds to time pressure:

*In my opinion, this represents a big discrepancy in terms of the systems not being implemented in a way that supports medicine […], ie, double and triple entry [of the same data].*

[interview 12, position 57, open code: user supports system no other way around]

### Regulations

Within the given context of Germany, the reimbursement of hospital services is only granted by the legislator if the documentation of services is seamless. However, since documentation requirements are increasingly more stringent, according to the interviewees, the regulations result in enormous efforts with data entry in the HIS, leading to adverse effects on interaction with the patients:

*Yes, well, it just keeps getting a lot. It is getting more and more; we have to document things that we did not have to in the past. I used to have more time for the patients, that is falling […] extremely short.*

[interview 2, position 19, open code: always increasing workload]

Consequently, the need to meet certain regulations puts further time pressure on medical staff, encouraging workarounds.

### Sector Funding

Furthermore, study participants highlighted aspects as relevant, regarding the funding of the sector:

*IT is an issue which, because it requires an investment on the largest scale, is linked to this overall catastrophic funding situation of public welfare.*

[interview 1, position 75, open code: resource allocation]

It is therefore perceived by the participants as their concerns with the HIS, and thus, the occurrence of workarounds can only be solved if appropriate funding is available.

### Role of Software Provider

The interviewees perceived the role of the individual software providers in the HIS economy as influential for the overall development and state of HIS. The participants perceive an absence of market conduct in the HIS industry:

*[HIS system] is the only supplier, the biggest one, and the other suppliers are much worse and are not subject to pressure. They have no need to implement innovation quickly and on time.*

[interview 5, position 57, open code: market conduct: oligopolistic structure hinders innovation]

Hence, the current market situation does not incentivize the software provider to optimize the software according to user needs and therefore encourages workarounds.

### Role of Ownership and Management

Budgetary decisions in terms of the employed staff, budgetary allocations in infrastructure, intrinsic motivation, or IT- and business strategy were reported to be influential for the occurrence of workarounds. For example, staff-to-patient ratios are often not calculated in such a way that a sufficient ratio of nurses toward the number of patients is achieved. Such stipulations can be crucial for the development of HIS-related workarounds:

*Well, in principle, the head nurses [with their additional managing workload] are also included as a regular part of the nursing staff. This means that they have to participate in the nursing work [just as much]. We are a small ward, consisting of seven full-time staff positions split between fourteen people. It is a pretty small amount [of staff] per shift.*

[interview 9, position 6, open code: nurse-to-patient-ratio]

## Discussion

### Key Findings

**Figure 4.**
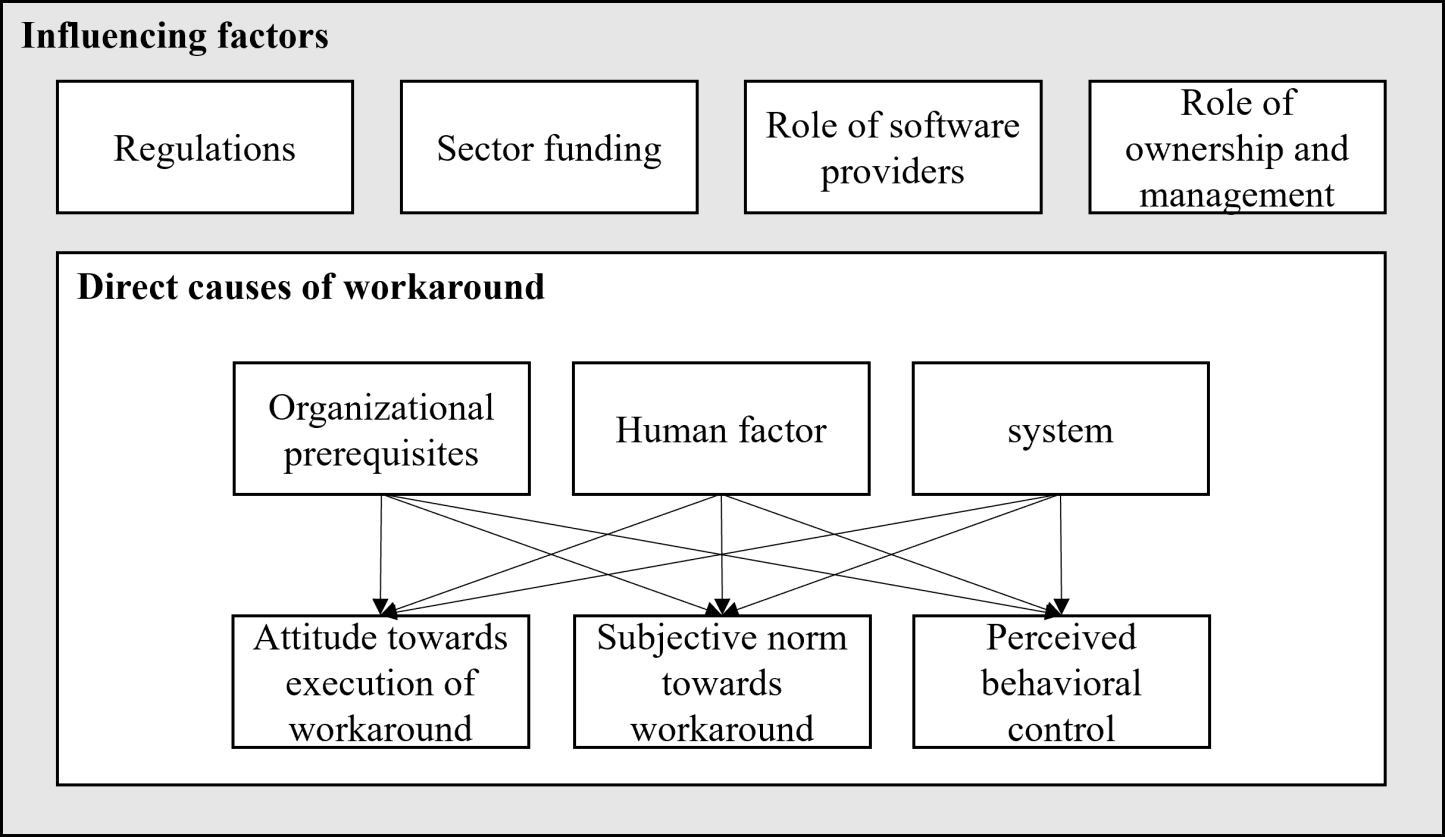
Model of antecedents of workarounds related to the hospital information system.

In the following, we discuss the identified antecedents of HIS-related workarounds, proposing a model that outlines the relationships between the identified antecedents of workarounds related to HIS (Figure 4).

With our study, we identified 3 direct causes (organizational prerequisites, human factor, and system) and 4 influencing factors (regulations, sector funding, role of software providers, and role of ownership and management) presenting the antecedents for HIS-related workarounds. Not all of the concepts we identified are new to the research stream; we also discovered similarities with existing results. For example, by identifying the concept of IT infrastructure (organizational prerequisites), we confirm the research of Lafferty et al [46] who examine the occurrence of workarounds related to the use of communication technologies such as pagers and phones and conclude that there is often a lack of IT equipment, in this case, mobile phones. The same issue is raised by Interviewee 2 who states that a lack of appropriate IT equipment, in our case workstations, means that medical staff often use the same accounts to bypass the slow login process (interview 2, position 15). Regarding the concept of skillset (human factors), we agree with Flanagen et al [47], who conclude that the users’ knowledge of how to use the electronic health record system influences the occurrence of workarounds. We are also in line with existing research regarding the concept of system interface (system). A complex order entry and difficulties in finding the needed data are also mentioned by Stevenson et al [48], who focus on the documentation problem of vital signs in the electronic health record system. Besides, we found similarities with those found by Bhattacherjee et al [49] regarding our category regulations. Strict documentation requirements lead to more time on the HIS system and less time in patient care, which causes the medical staff to commit workarounds to avoid patient care being compromised (interview 2, position 19) [49]. The system category involves HIS restrictions from either deliberate restrictions implemented in the HIS’s design or limitations in functionality, as perceived by users [50].

Besides highlighting similarities, we expand the literature in the context of HIS-related workarounds by revealing the further antecedents: sector funding, role of software provider, role of ownership and management, IT support (organizational prerequisites), patient demands (human factor), functionalities (system), and user involvement in design (system). With sector funding*,* we involve a monetary component. Without sufficient financial resources, certain challenges (eg, insufficient IT equipment and support) emerge in the first place (interview 1, position 84). The role of software providers stands apart as novel as their oligopolistic influence is very specific to the hospital market (interview 5, position 57). We further contribute to the role of ownership and management, as their operational decisions and organizational values shape the daily operations (eg, insufficient staffing) (interview 2, position 13). Further, we were able to add to the existing knowledge with organizational prerequisites, namely the concept of IT support. Insufficient staffing levels and organizational background in hospital IT departments dominate the emergence of workarounds, as users are left unsupervised to a large extent (interview 2, position 63). Regarding the human factor, we were able to add the concept of patient demands as antecedent, which distinctly characterizes the personal relations and expectations within medical service delivery and how meeting these demands affects the emergence of workarounds (interview 5, position 45; Interview 4, position 257). Further discovered antecedents of HIS-related workarounds lie in the system, precisely in the concepts of functionalities and user involvement in design. In terms of functionalities, we extend the current knowledge by highlighting the missing functionality of shared editing between multiple users within the HIS as an antecedent for workarounds. User involvement in design highlights the importance of integrating medical and procedural knowledge into system design, which is now dominated by nonmedical developers (interview 5, position 51). These concepts emphasize just how important it is that technology facilitates medical service delivery with its interprofessional and interdisciplinary nature and must therefore be completely aligned with the needs of the user, as otherwise, workarounds may appear necessary.

Besides confirming existing research and identifying new antecedents from a static perspective, we revealed relationships between them. In the following, we illustrate the proposed relationships between direct causes and the TPB components using concept-level examples. Our assumptions are grounded in the interview data and supported by existing literature. We also specify the intention to benefit that may drive the *Direct Cause* in each example and include relevant influencing factors*.*

Our proposed model highlights rationales that are antecedents to the constructs underlying the TPB. Thereby, influencing factors, in our study identified as regulations, sector funding, role of software providers, and role of ownership and management, serve as second-level denominators in the research endeavor of explaining antecedents of workarounds related to HIS. As they constitute higher-level influences, they do not directly impact nurses’ and physicians’ behavior but rather depict the defaults that lead to conditions for direct causes of workarounds. Human factors, systems, and organizational prerequisites form the direct causes of workarounds. These direct causes impact the attitude towards the execution of workaround, the subjective norm towards workaround, and the perceived behavioral control.

Exemplarily, insufficient IT infrastructure (organizational prerequisites) is reported to negatively influence users’ attitudes, as demonstrated, for example, by Cresswell et al [51], who investigate the adoption of large-scale health information technology. In line with this, our results indicate that queuing in front of IT devices can lead users to resort to workarounds (interview 2, position 41). Underlying is the assumption that a workaround is a solution to the acute problem, which leads to a positive attitude toward the execution of the workaround with the intention to benefit the users themselves (by avoiding waiting time) and to benefit the patient (as this time can be spent on care). Hence, we link organizational prerequisites to attitude towards workarounds. An influencing factor on the organizational prerequisites is, for example, the overall sector funding, which leads to a limited IT budget and insufficient equipment. Regarding the physician-patient relationship (human factor), we align with Buck et al’s [42] notion of the influence on attitude. They investigate general practitioners’ attitudes towards the use of artificial intelligence in medical diagnosis and conclude that physicians are concerned about a possible impairment of the physician-patient relationship when technology is used. We argue that this comparable result can help to concretize the effect of human factors on attitude toward workarounds*.* The human factor itself is conditioned by, for example, the influencing factor regulation, as documentation requirements in the hospital lead to less time for patient interaction. This may encourage the user of HIS to view a workaround as a solution with the intention to benefit the patient with greater attention and handle the system later (interview 8, position 59). In another notion, Beereport et al [50] include instances of workarounds where the same professional actors have power over the same type, eg hierarchy: lead physicians over junior physicians. Further, they include power dynamics across different actor types, eg physicians over nurses. This extends our results to the human factor’s power dynamics being one standalone source of workarounds. The influence of ease of use and intuitivity (system), for example, on attitude is featured in Van Schaik et al’s [52] adapted Technology Acceptance Model for computerized physician order entry systems in primary care. The system design features influence the perceived ease of use and perceived usefulness and therefore the attitude toward using IT. We argue that the attitude towards using HIS is the inverse of the attitude towards workaround behavior related to HIS [32], suggesting a relationship between the system and attitude towards the execution of workaround in our model. In this regard, it is emphasized that linking formal and informal information systems offers a comprehensive view of boundary infrastructures, workarounds, and shadow systems to address gaps in information needs [53]. Some limitations stem from the design choices made by the HIS suppliers, while others are a result of the specific configurations implemented by the organization’s IT department [50]. One influencing factor on the system is the role of software providers*,* who, due to their oligopoly position, lack the incentive to tailor the HIS to the users’ needs. Because it is not user-friendly and requires time-consuming processes, medical staff avoids tedious tasks related to the HIS. This often leads to the development of workarounds primarily with the intention to benefit the medical staff.

Research on normative conduct suggests that visibility of the behavior of the social surroundings can be an effective means of influencing social norms and inducing individual behavior change [54]. We apply this to form a relationship between organizational prerequisites and subjective norms towards workarounds since medical emergencies (characteristics of medical care) require fast responses, and patterned workarounds can reduce the cognitive effort required to deal with each new emergency [55], with the intention to benefit the patient. Regarding the influences of human factors, we draw on Stacey et al [56], who describe how differing expectations between patients and physicians create a demanding encounter. Expectations are placed on medical providers, especially in terms of maintaining an intense physician-patient relationship and educating the patient. Applied to our findings, the patient demands (human factor) affect the subjective norms towards workarounds when these expectations from individuals put pressure on the group of health care providers. This favors workarounds to meet patient demands as the “new normal” (with the intention to benefit the patient). We further argue that, particularly concerning the system, colleagues can relate to individual users’ experiences with nonfitting HIS functionalities and perceived blockages. From the common frustration and knowledge sharing on workarounds, subjective norms can emerge that legitimize and, thus, favor the occurrence of workarounds [57,58] with the intention to benefit oneself as well as the local unit (ward). Thus, we link system and subjective norms towards workarounds.

In terms of perceived behavioral control, lacking or low supervision relates to the ease of workaround performance, consisting of the process design, system access control, and unclear or undefined roles [7]. When these structures support or hinder certain actions, they shape individuals’ perceived behavioral control by either facilitating or restricting their perceived ability to perform specific behaviors. Hence, we link organizational prerequisites to perceived behavioral control. Existing research has identified that guideline adherence and hierarchical influences limit the perceived area of action. Possible reasons are, for example, cultural or generational values that emphasize “sticking to the rules” or insecurities, such as when desiring to “fit in” [59]. Therefore, we link individual character traits (human factors) to perceived behavioral control*.* A dominant influence on perceived behavioral control is rooted in the Hippocratic oath, which postulates the core value of serving sick people and humanity, prioritizing ethics and patients’ health. Working under this oath gives physicians strong behavioral control over their workaround intention and behavior, as they act with ethical backing [60]. Currie et al [61] highlight that institutional structures often prioritize the autonomy and authority of medical specialists over other groups. In response, those under such power may develop workarounds to challenge or bypass imposed systems and rules [62]. Similarly, systemic power is evident when users adapt technology to suit their own needs and interests. With these various perspectives, we engage existing literature on perceived behavioral control to enrich the proposed relationship between human factors and perceived behavioral control. Regarding the link between the system and perceived behavioral control, IS can restrict users’ work practices through the procedural constraints embedded within them [63]. This creates a situation similar to bureaucratic control, where users—such as physicians, nurses, and other staff—are initially constrained by the IS’s episodic power [50]. However, users often respond by exerting their own power through workarounds, which can be seen as a way to break free from this “iron cage” [64]. Contrary to the view that users are merely trapped by IS design, these workarounds demonstrate that users are active agents, adapting the IS to better fit their needs [50]. This process empowers users and enhances their perceived behavioral control, as they realize their capacity to effect change despite systemic constraints.

### Theoretical Contribution and Practical Implications

Our research makes a valuable theoretical contribution by applying the TPB to the context of workarounds related to HIS, thus providing a detailed understanding of their behavior. By identifying both the direct causes and influencing factors that affect attitudes towards workarounds, subjective norms towards workarounds, and perceived behavioral control, we have strengthened the explanatory power of the TPB. Our research identifies the specific causes and influences of workarounds within the TPB framework, which contextualizes the theory, detailing and applying it, ultimately leading to improved predictive power. Furthermore, we add to the existing body of knowledge by confirming existing novel factors and adding selective ones. Our findings also contribute to the wider literature on workarounds, deviations, and human error, as they highlight the importance of considering not only individual factors but also broader contextual factors that shape behavior. In this regard, we sort our research into the theory of workarounds [5], specifically, the steps intentions, goals, interests, structure, and perceived need for a workaround and provide details for the health care sector. This deepened understanding can inform the development of interventions and strategies to prevent the need for workarounds, ultimately improving patient safety and the overall quality of health care. Further, we support the extension of the TPB by Soffer et al [7], who distinguish local-unit goals from organizational goals and include their intention to benefit in explaining behavior, in the context of patient care. While Soffer et al [7] analyze goal-driven behavior and unfold external factors in an organizational context, we study patient care, where workarounds are performed to promote the well-being of patients, unlike other contexts, where they may be done for personal gain. This is in accordance with Patterson [65], who describes goal trade-offs that occur when one task is delayed due to competing demands or when tasks are performed in an unintended sequence. This situation highlights the need for organizational priorities, which, in this context, also include ethical considerations. Therefore, it is critical to consider the intention to benefit as an essential factor in evaluating behaviors in patient care and to acknowledge the valuable contribution made by Soffer et al [7] in this area.

Besides theoretical contributions, we also derived valuable practical implications. By identifying the direct causes and influencing factors of workarounds, we help to better understand the behavior of medical staff and develop strategies to mitigate root causes, thus, the emergence of HIS-related workarounds. Directly involved stakeholders, such as users, HITs, software developers, and managers, can address direct causes. Health care organizations can draw on these findings to implement targeted measures to improve working conditions. Our findings suggest that the IT infrastructure, such as a consistently stable internet connection, and IT equipment, such as a sufficient number of workstations, are significant to the emergence of workarounds related to HIS. Health care organizations need to invest in better IT equipment and IT infrastructure to reduce the frequency of workarounds motivated by these factors. Investing in a high-speed internet connection can also minimize frustration and time pressure. Further, our research findings can also serve as an impetus for health care organizations to develop educational materials and training programs for medical staff to improve their skills and knowledge in dealing with HIS, thereby building knowledge that enhances system use and feedback. Second, our article provides implications for software developers, as it identifies system-related antecedents for workarounds related to HIS, such as missing interfaces to other systems, missing functionalities, and difficulties with system usability. By incorporating these findings into the improvement of HIS, software developers can enhance the system’s functionality and usability, ultimately reducing the occurrence of workarounds. Additionally, we recommend involving end users, medical staff, in the development process to ensure that the HIS meets their needs and is user-friendly. This approach can lead to the development of a more efficient and effective HIS, benefiting both medical staff and patients. Above that, politics, self-administration in health care, and associations can address influencing factors, thereby altering the framework conditions of medical service provisions. To support the user-centered improvement of HIS, political action is necessary. The current oligopolistic situation of HIS providers on the market, and the resulting lack of incentives for HIS providers to make improvements, can be addressed through political action. By promoting competition and innovation, policy makers can incentivize HIS providers to improve their products and services, leading to a better HIS that meets the needs of medical staff and patients.

### Limitations

While adhering to a consistent research approach, our study is subject to limitations, in part related to our methodological choice of qualitative exploratory interviews. First, qualitative interviews are generally not designed to draw holistic conclusions, which compromises the generalizability of the findings. However, the interviews allowed us to gain a deeper understanding of the nurses’ and physicians’ behavior and the complexity of their personal motivation for evasion. In addition, by having direct contact with the medical staff, we were able to identify the interplay and cause-effect relationships of the identified antecedents. Second, it is important to acknowledge that our study relied on self-reported data from the interview participants. Self-reporting can introduce biases, as participants may not fully disclose all relevant behaviors or may present themselves in a more favorable light, particularly when discussing sensitive topics like circumventing regulations [66]. This limitation could potentially lead to an incomplete understanding of the underlying reasons for workaround behaviors. To mitigate this potential risk of the incompleteness of workarounds’ antecedents, we built rapport and trust with our interview participants, provided assurance, and emphasized the confidentiality of their responses by distancing ourselves from management [38]. We acknowledge that our study could have been further strengthened by incorporating triangulation methods, such as observational data, to complement the self-reported interviews [67]. Similarly, system feature assessments and tracking data could provide quantifiable evidence of HIS use patterns and workaround behaviors, offering a valuable cross-reference to the qualitative findings [68]. The third limitation of our study is the brevity of some interviews due to them being conducted during off-peak times, such as before or after shifts or during lunchtime, where emergent work commitments occasionally required participants to end the interview early. Although these interviews were included in the analysis for their consistency with earlier findings, the reduced time may have limited the depth of information collected. Fourth, the relationships between influencing factors and direct causes on attitude towards the execution of workaround, the subjective norm towards workaround, and the perceived behavioral control are not quantitatively tested and validated, creating directions for further research. However, for this study, we have used existing literature to strengthen the assumptions regarding the relationships. Similarly, the scope of this study is limited to identifying the antecedents of workaround behavior, without delving into the cause-effect relationships between these antecedents and specific workarounds (and their analysis) used by medical personnel.

### Conclusion and Future Research

As hospitals face mismatches between HIS design, workflows, and user requirements, workaround behavior seems inevitable for nurses and physicians to overcome perceived hurdles of HIS and still serve the patients. This paper provides a deeper understanding of the antecedents of HIS-related workarounds, laying the foundation for addressing underlying issues and reducing the need for workarounds. By conducting 26 interviews with nurses, physicians, and HITs and analyzing the interviews using grounded theory techniques, we identified 18 antecedents of HIS-related workarounds, which we clustered into 3 direct causes (organizational prerequisites, human factor, and system) and 4 influencing factors (regulations, sector funding, role of software providers, and role of ownership and management).

We present a comprehensive model and theoretically embed our findings into the TPB, as this allows for the explanation of why an intention to execute workaround behavior is formed. Thereby, we reveal that attitude towards the execution of workaround, subjective norm towards workaround, and perceived behavioral control are influenced by direct causes*,* which in turn are affected by the influencing factors. We incorporate the notion that workarounds are executed with the intention to benefit either oneself, the patient, the local unit (ward), or the organization. We confirm and extend previous research on the antecedents of workarounds related to HIS by adding the influencing factors of sector funding, role of software provider, and role of ownership and management. Further, we incorporate the previously unexplored antecedents of user involvement in design, patient demands*,* and IT support. Our findings offer valuable insights on 2 levels: Nurses and physicians, HITs, hospital managers, and software developers can potentially impact the antecedents of direct causes, while politics and associations can adjust the framework conditions (influencing factors) in which medical service provision takes place. Addressing both levels can improve working conditions, patient safety, and the overall quality of health care.

The obtained results should be interpreted against the background of the two study locations, Germany and the United States. In addition to common reference points, the sample revealed structural differences that can affect organizational performance and, thereby, the execution of workarounds. The resource allocation of the two countries concerning IT in hospitals represents a very different baseline. The financial situation and, accordingly, the investment budget as well as the staffing ratios in the considered US hospital were reported to be much higher than the situation of the (publicly funded) participating German hospitals. In particular, the existing staffing ratios on the hospital floors and IT departments were described as influential on the behavior of the participants since they led to a different use of the system. This is what enables reflection on the HIS in a structured way, as well as comprehension and use so that practical improvements can be proposed. The development of trusting feedback mechanisms provides an important starting point for future research, as workarounds can be taboo subjects, and it is vital to find sensible ways of breaking down any reluctance in sharing feedback. The participating US American hospital served as a pilot hospital in the development of the HIS, having entered into a cooperation agreement with the HIS vendor, which resulted in stronger IT and vendor support. This setting factor explicitly shows the importance of feedback and feedback handling for the design of the system. We, therefore, call for further research to elaborate on structural differences between countries within the context of the emergence of workarounds.

The COVID-19 pandemic heavily impacted workflows and routines, the involved staff, and the patients. Examples are the enormous workload caused by patient volumes, emotional distress through the confrontation with death, and changes in hygiene regulations [69]. Therefore, we argue that these shifts in priorities potentially altered the attitude towards workarounds. In order to better prepare for future crises, prevent workarounds with harmful consequences, and build resilience, more research on the influence of crisis situations on workaround intention is needed.

## Supplementary material

10.2196/51781 Multimedia Appendix 1 Table containing examples from the coding catalog.

10.2196/51781 Checklist 1 Standards for Reporting Qualitative Research (SRQR).

## Abbreviations

GTM: grounded theory methodology
HIS: hospital information system
HIT: health information technician
TPB: theory of planned behavior

## References

[1] Georgantzas NC, Katsamakas EG (2008). Information systems research with system dynamics. Syst Dyn Rev.

[2] Mehdipour Y, Zerehkafi H (2013). Hospital information system (his): at a glance. Asian J Comput Inf Syst.

[3] Eason K, Waterson P (2014). Fitness for purpose when there are many different purposes: who are electronic patient records for?. Health Informatics J.

[4] Beglaryan M, Petrosyan V, Bunker E (2017). Development of a tripolar model of technology acceptance: hospital-based physicians’ perspective on EHR. Int J Med Inform.

[5] Alter S (2014). Theory of workarounds. CAIS.

[6] Ejnefjäll T, Ågerfalk PJ (2019). Conceptualizing workarounds: meanings and manifestations in information systems research. CAIS.

[7] Soffer P, Outmazgin N, Hadar I, Tzafrir S (2023). Why work around the process? analyzing workarounds through the lens of the theory of planned behavior. Bus Inf Syst Eng.

[8] Niazkhani Z, Pirnejad H, van der Sijs H, Aarts J (2011). Evaluating the medication process in the context of CPOE use: the significance of working around the system. Int J Med Inform.

[9] Strong D, Volkoff O (2010). Understanding organization—enterprise system fit: a path to theorizing the information technology artifact. MIS Q.

[10] Boudreau P, Vieru D, Paquette G, Heon M The workarounds process as a source of knowledge creation and management.

[11] McGann S, Lyytinen K The improvisation effect: a case study of user improvisation and its effects on information system evolution. https://dblp.org/rec/conf/icis/McGannL08.

[12] Röder N, Wiesche M, Schermann M, Krcmar H (2014). Why managers tolerate workarounds: the role of information systems. https://scholarcommons.scu.edu/omis/21/.

[13] Boonstra A, Jonker TL, van Offenbeek MAG, Vos JFJ (2021). Persisting workarounds in electronic health record system use: types, risks and benefits. BMC Med Inform Decis Mak.

[14] van der Veen W, Taxis K, Wouters H (2020). Factors associated with workarounds in barcode-assisted medication administration in hospitals. J Clin Nurs.

[15] Eikey EV, Chen Y, Zheng K (2019). Cognitive Informatics.

[16] Rathert C, Porter TH, Mittler JN, Fleig-Palmer M (2019). Seven years after meaningful use: physicians’ and nurses’ experiences with electronic health records. Health Care Manage Rev.

[17] Blijleven V, Hoxha F, Jaspers M (2022). Workarounds in electronic health record systems and the revised sociotechnical electronic health record workaround analysis framework: scoping review. J Med Internet Res.

[18] Ajzen I (1991). The theory of planned behavior. Organ Behav Hum Decis Process.

[19] Eisenhardt KM (1989). Agency theory: an assessment and review. Acad Manage Rev.

[20] Baker T, Nelson RE (2005). Creating something from nothing: resource construction through entrepreneurial bricolage. Adm Sci Q.

[21] Engeström Y, Miettinen R, Punamäki RL (1999). Perspectives on Activity Theory.

[22] Dacin MT, Goodstein J, Scott WR (2002). Institutional theory and institutional change: introduction to the special research forum. AMJ.

[23] Orlikowski WJ, Gash DC (1994). Technological frames: making sense of information technology in organizations. ACM Trans Inf Syst.

[24] Rogers Y Exploring obstacles: integrating CSCW in evolving organisations.

[25] Hagger MS, Chatzisarantis NLD, Biddle SJH (2002). A meta-analytic review of the theories of reasoned action and planned behavior in physical activity: predictive validity and the contribution of additional variables. J Sport Exerc Psychol.

[26] Riebl SK, Estabrooks PA, Dunsmore JC (2015). A systematic literature review and meta-analysis: the theory of planned behavior’s application to understand and predict nutrition-related behaviors in youth. Eat Behav.

[27] Apau R, Koranteng FN (2020). Impact of cybercrime and trust on the use of e-commerce technologies: an application of the theory of planned behavior. Int J Cyber Criminol.

[28] Campbell EM, Guappone KP, Sittig DF, Dykstra RH, Ash JS (2009). Computerized provider order entry adoption: implications for clinical workflow. J Gen Intern Med.

[29] Campbell EM, Sittig DF, Ash JS, Guappone KP, Dykstra RH (2006). Types of unintended consequences related to computerized provider order entry. J Am Med Inform Assoc.

[30] Saleem JJ, Russ AL, Neddo A, Blades PT, Doebbeling BN, Foresman BH (2011). Paper persistence, workarounds, and communication breakdowns in computerized consultation management. Int J Med Inform.

[31] Yang Z, Ng BY, Kankanhalli A, Luen Yip JW (2012). Workarounds in the use of IS in healthcare: a case study of an electronic medication administration system. Int J Hum Comput Stud.

[32] Buck C, Doctor E, Eymann T, Simoes EJ A systematic literature review on antecedents of workarounds related to information systems in hospitals.

[33] Schultze U, Avital M (2011). Designing interviews to generate rich data for information systems research. Information and Organization.

[34] Myers MD, Newman M (2007). The qualitative interview in IS research: examining the craft. Information and Organization.

[35] Kaplan B, Maxwell JA, Anderson JG, Aydin CE (2005). Evaluating the Organizational Impact of Healthcare Information Systems.

[36] Mey G, Mruck K, Naderer G (2007). Qualitative Marktforschung in Theorie Und Praxis: Grundlagen, Methoden Und Anwendungen.

[37] Corbin JM, Strauss AL (2008). Basics of Qualitative Research: Techniques and Procedures for Developing Grounded Theory.

[38] Bergen N, Labonté R (2020). “Everything is perfect, and we have no problems”: detecting and limiting social desirability bias in qualitative research. Qual Health Res.

[39] Creswell JW (2009). Research Designs: Qualitative, Quantitative, and Mixed Methods Approaches.

[40] Corbin J (2008). Basics of Qualitative Research: Techniques and Procedures for Developing Grounded Theory.

[41] Nelson J (2017). Using conceptual depth criteria: addressing the challenge of reaching saturation in qualitative research. Qual Res.

[42] Buck C, Doctor E, Hennrich J, Jöhnk J, Eymann T (2022). General practitioners’ attitudes toward artificial intelligence-enabled systems: interview study. J Med Internet Res.

[43] Kallio H, Pietilä AM, Johnson M, Kangasniemi M (2016). Systematic methodological review: developing a framework for a qualitative semi-structured interview guide. J Adv Nurs.

[44] Glaser BG, Strauss AL (1967). The Discovery of Grounded Theory.

[45] O’Brien BC, Harris IB, Beckman TJ, Reed DA, Cook DA (2014). Standards for reporting qualitative research: a synthesis of recommendations. Acad Med.

[46] Lafferty M, Harrod M, Krein S, Manojlovich M (2021). It’s like sending a message in a bottle: a qualitative study of the consequences of one-way communication technologies in hospitals. J Am Med Inform Assoc.

[47] Flanagan ME, Saleem JJ, Millitello LG, Russ AL, Doebbeling BN (2013). Paper- and computer-based workarounds to electronic health record use at three benchmark institutions. J Am Med Inform Assoc.

[48] Stevenson JE, Israelsson J, Nilsson G, Petersson G, Bath PA (2018). Vital sign documentation in electronic records: the development of workarounds. Health Informatics J.

[49] Bhattacherjee A, Davis CJ, Connolly AJ, Hikmet N (2018). User response to mandatory IT use: a coping theory perspective. Eur J Inf Syst.

[50] Beerepoot I, Koorn J, Weerd I, Hooff B, Leopold H, Reijers HA Working around health information systems: the role of power. https://research.vu.nl/en/publications/working-around-health-information-systems-the-role-of-power.

[51] Cresswell KM, Bates DW, Sheikh A (2013). Ten key considerations for the successful implementation and adoption of large-scale health information technology. J Am Med Inform Assoc.

[52] Van Schaik P, Flynn D, Van Wersch A, Douglass A, Cann P (2004). The acceptance of a computerised decision-support system in primary care: a preliminary investigation. Behav Inf Technol.

[53] Mörike F, Spiehl HL, Feufel MA (2024). Workarounds in the shadow system: an ethnographic study of requirements for documentation and cooperation in a clinical advisory center. Hum Factors.

[54] Cialdini RB, Kallgren CA, Reno RR (1991). A focus theory of normative conduct: a theoretical refinement and reevaluation of the role of norms in human behavior. Adv Exp Soc Psychol.

[55] Kobayashi M, Fussell SR, Xiao Y, Seagull FJ Work coordination, workflow, and workarounds in a medical context.

[56] Stacey CL, Henderson S, MacArthur KR, Dohan D (2009). Demanding patient or demanding encounter?: a case study of a cancer clinic. Soc Sci Med.

[57] Varpio L, Schryer CF, Lehoux P, Lingard L (2006). Working off the record: physicians’ and nurses’ transformations of electronic patient record-based patient information. Acad Med.

[58] Zuzelo PR, Gettis C, Hansell AW, Thomas L (2008). Describing the influence of technologies on registered nurses’ work. Clin Nurse Spec.

[59] Jones A, Blake J, Adams M, Kelly D, Mannion R, Maben J (2021). Interventions promoting employee “speaking-up” within healthcare workplaces: a systematic narrative review of the international literature. Health Policy.

[60] Burns AJ, Young J, Roberts T (2015). Exploring the role of contextual integrity in electronic medical record (EMR) system workaround decisions: an information security and privacy perspective. THCI.

[61] Currie G, Lockett A, Finn R, Martin G, Waring J (2012). Institutional work to maintain professional power: recreating the model of medical professionalism. Organization Studies.

[62] Doolin B (2004). Power and resistance in the implementation of a medical management information system. Information Systems Journal.

[63] Gosain S, University of Maryland, USA (2004). Enterprise information systems as objects and carriers of institutional forces: the new iron cage?. JAIS.

[64] Huuskonen S, Vakkari P (2013). “I Did It My Way”: social workers as secondary designers of a client information system. Inf Process Manag.

[65] Patterson ES (2018). Workarounds to intended use of health information technology: a narrative review of the human factors engineering literature. Hum Factors.

[66] Podsakoff PM, MacKenzie SB, Lee JY, Podsakoff NP (2003). Common method biases in behavioral research: a critical review of the literature and recommended remedies. J Appl Psychol.

[67] Carter N, Bryant-Lukosius D, DiCenso A, Blythe J, Neville AJ (2014). The use of triangulation in qualitative research. Oncol Nurs Forum.

[68] Zheng K, Padman R, Johnson MP, Diamond HS (2009). An interface-driven analysis of user interactions with an electronic health records system. J Am Med Inform Assoc.

[69] Dinibutun SR (2020). Factors associated with burnout among physicians: an evaluation during a period of COVID-19 pandemic. J Healthc Leadersh.

