# Supplementary material for: Understanding Antecedents of Nurses' and Physicians' Workaround Behavior Regarding Hospital Information Systems: Qualitative Interview Study
Source: J Med Internet Res. 2025 Jul 15;27:e51781. doi: 10.2196/51781 (PMC12282938; doi:10.2196/51781)
Supplement: Multimedia Appendix 1 [file jmir-v27-e51781-s001.docx]

Table S1. Example from the coding catalog.

| **Code name** | **Definition** | **Example** | **Coding rule** |
| --- | --- | --- | --- |
| Designed by non-medical staff, thus misfit | Refers to the participants’ perception that HIS are not designed from the end-user’s perspective and therefore do not meet their needs. | “Well, the systems are usually created by people who have never done the work themselves.” [interview 12, position 37] | This code includes perceptions that HIS are not designed for the end users’ need. |
