# Supplementary material for: Understanding Antecedents of Nurses' and Physicians' Workaround Behavior Regarding Hospital Information Systems: Qualitative Interview Study
Source: J Med Internet Res. 2025 Jul 15;27:e51781. doi: 10.2196/51781 (PMC12282938; doi:10.2196/51781)
Supplement: Checklist 1 [file jmir-v27-e51781-s002.pdf]

Standards for Reporting Qualitative Research (SRQR)

Title and abstract

	**Page/line no(s).**
**Title** - Concise description of the nature and topic of the study Identifying the study as qualitative or indicating the approach (e.g., ethnography, grounded theory) or data collection methods (e.g., interview, focus group) is recommended<br><br>*Understanding Nurses' and Physicians' Workaround Behavior Regarding Hospital Information Systems: Qualitative Interview Study*	p. 1
**Abstract** - Summary of key elements of the study using the abstract format of the intended publication; typically includes background, purpose, methods, results, and conclusions<br><br>*Background: Hospital Information Systems (HIS) aim to support users in their time-critical routines on hospital wards with accurate and timely information. However, if these systems create blockages to workflows, nurses and physicians develop workarounds to provide care to the patients, nonetheless. Workarounds are both considered negatively, when associated with risks, and positively, when seen as feedback and source of innovation. Learning about the antecedents of workarounds allows for the establishment of control mechanisms, under the promise of enhanced patient safety.*<br>*Objective: This study seeks to explore which antecedents shape nurses' and physicians' workaround behavior in the context of HIS, how they influence behavior and interrelate, along with the intentions with which they are carried out.*<br>*Methods Utilizing 26 qualitative interviews with nurses, physicians, and health in-formation technicians from Germany and the USA and applying grounded theory analysis techniques, we identify antecedents of HIS-related workarounds and respective relations.*<br>*Results: From the interview transcripts, we derive 506 open codes, which we merge cluster into three Direct Causes (Organizational Prerequisites, Human Fac-tor, System), and four Influencing Factors (Regulations, Sector Funding, Role of Software Providers, Role of Ownership and Management). While Influencing Factors constitute higher-level influences, they do not directly impact nurses' and physicians' behavior but rather depict the defaults that lead to conditions for Direct Cause*s of workarounds.<br>Conclusions: This study provides an understanding of the antecedents of workarounds performed by medical personnel regarding HIS usage, structures and categorizes them, and lays the foundation for an understanding of users' deviant behavior. Moreover, by revealing cause-effect relationships between the antecedents, we take on a behavioral perspective and provide a basis for developing effective strategies to prevent the need for workarounds. We contribute to the research stream of workarounds in healthcare and emphasize that once the reported and derived Direct Causes and Influencing Factors of workarounds have been tackled, working conditions, patient safety, and the overall quality of healthcare may improve under full digital support.	p. 1

Introduction

**Problem formulation** - Description and significance of the problem/phenomenon studied; review of relevant theory and empirical work; problem statement<br><br>*Even though HIS promise to support the medical staff, in reality, it is often otherwise, and they are rather perceived as a hindrance than a support.*<br>*When HIS are perceived as obstructive, they do not depict nurses' and physicians' workflows, forcing them to engage in coping behavior – so-called workarounds [3].*<br>*This off-track behavior is widespread, often consisting of a set of work practices not formally described in process models, rules, and regulations, such as omitting or adding steps and performing unauthorized actions to still reach a certain goal.*<br>*Whenever workarounds occur, they serve as indicators for a mismatch between the involved information systems, defined processes, and user requirements [8].*<br>*The potential adverse effects of workaround behavior in patient care underscore the criticality of understanding the antecedents of their occurrence.*	p. 2
**Purpose or research questio**n - Purpose of the study and specific objectives or questions<br><br>*The purpose of this study is to examine how underlying antecedents shape individual workaround behavior in the context of HIS. Thereby, we incorporate cognitive, social, and contextual elements, take on a cause-effect perspective, and emphasize the complexity of medical personnels' intentions when engaging in workaround behavior. Unrevealing this knowledge can bring attention to working conditions, facilitate HIS and workflow redesign, and push the development of more fitting guidelines.*	p. 5

Methods

**Qualitative approach and research paradigm** - Qualitative approach (e.g., ethnography, grounded theory, case study, phenomenology, narrative research) and guiding theory if appropriate; identifying the research paradigm (e.g., postpositivist, constructivist/ interpretivist) is also recommended; rationale**<br><br>*This study follows the qualitative research approach and interpretivist paradigm, uses grounded theory as the methodological approach, especially the three-step Straussian approach for data analysis (Corbin und Strauss 2014). The rationale is to obtain in-depth, real-world depictions from frontline users and capture detailed and deep information to understand nurses' and physicians' behavior, emphasizing the complexity of process design and personal motivation.*	p. 5-6
**Researcher characteristics and reflexivity** - Researchers' characteristics that may influence the research, including personal attributes, qualifications/experience, relationship with participants, assumptions, and/or presuppositions; potential or actual interaction between researchers' characteristics and the research questions, approach, methods, results, and/or transferability<br><br>*The author team consists of two doctoral candidates of business informatics in the healthcare sector in their final year of the PhD program with a focus on qualitative studies, one scholar, and one senior scholars of business informatics with a focus on qualitative studies and Design Science Research. Each author team member has own experiences with the healthcare sector, for the doctoral candidates, it is personal experience from internships on hospital wards and own HIS usage during their university education, which may foster individual bias in terms of own experiences of workarounds (confirmation bias). For the scholars, it is in the form of research papers, which they supervised, here we expect no biases. To counteract*	n/a

any presuppositions, the authors engaged in constant comparison of interpretation and reflected discussions among themselves.	
**Context** - Setting/site and salient contextual factors; rationale**	
**Sampling strategy** - How and why research participants, documents, or events were selected; criteria for deciding when no further sampling was necessary (e.g., sampling saturation); rationale**<br><br>*We selected and recruited participants via convenience sampling, paying attention to a representation of age groups, gender distribution, levels of work experience and different HIS capabilities (determined by a guided self-estimation). We contacted individuals directly, as well as with an approach of contacting C-level management with email requests that provided a short description of the research purpose and further relied on personal network contacts. Additionally, we asked participants about colleagues that could be interested in participation. This sampling strategy allowed us to integrate participants that were open to talk about their behavior and perception willingly, as other, e.g., top-down approaches including management recommendations, would be counterproductive in this sensible domain. Data saturation with this sample was achieved for the research question under light, following Nelson (2017) in their notion of there being enough information to replicate the study and saturation in terms of additional new information in each data collection round, and when further coding for this topic is no longer feasible. Following our iterative process, we reviewed every round of additional data collection carefully, as we were coding in a timely manner after collecting the data. As we noticed significant duplicate mentions in the last four interviews, we decided to end the iterative process with a total of 26 interviews.*	p. 5-6
**Ethical issues pertaining to human subjects** - Documentation of approval by an appropriate ethics review board and participant consent, or explanation for lack thereof; other confidentiality and data security issues<br><br>*As we pseudonymized the collected data, no drawbacks are possible, and the anonymity of the participants is guaranteed. Further, all participants agreed to sign a consent form for data collection, analysis, and storage. By focusing on the individuals in their role as professional medical practitioners, we clearly refrain from taking a possible organizational perspective (e.g., by strongly distancing ourselves from management as neutral researchers). In this approach, we emphasize the individuals' own perception and their role as medical practitioners with all values and refrain from taking a managerial standpoint, e.g., we do not analyze data separately for the participating organizations or derive recommendations for each organization but aggregate the results.*	p. 5-6, p. 22
**Data collection methods** - Types of data collected; details of data collection procedures including (as appropriate) start and stop dates of data collection and analysis, iterative process, triangulation of sources/methods, and modification of procedures in response to evolving study findings; rationale**<br><br>*The data collection process involved a series of ongoing and repeated steps that worked together harmoniously. The collected data stems from semi-structured interviews, which were held face-to-face and via telephone. The collected data therefore represents audio files, which we transcribed using the software f4analyse*	p. 5-6

and coded using MaxQDA. The data collection process was iterative, meaning there is no direct sequence to the steps of collection and analysis, it is an ongoing effort. We started data collection in November 2019 and ended in March 2020. While employing the GTM and adhering to the three-step Straussian approach encompassing open, axial, and selective coding, we began by breaking the qualitative datasets down into relevant fragments (open coding). After an initial read of the transcripts, we highlighted phrases that were relevant to the research topic, resulting in 506 open codes. Following GTM techniques [43], we examined the codes and merged common topics into concepts. After ensuring an even allocation and hierarchy of the concepts, we merged them into categories and identified relationships (axial coding). Thereby, we distinguished the core category antecedents to HIS-related workarounds from other categories (selective coding) [39]. In order to validate the coding results, two authors performed a card-sorting allocation. The first author identified open codes and concepts, which served as the basis for the second author to conduct a blind card-sorting round. The second author added not initially identified open codes along the process. Whenever there were discrepancies in the allocations of the authors, the authors discussed the results to find agreements. The coding process was iteratively refined through constant comparison and adjustments, and the open coding steps were repeated backward and forward whenever new insights were gained [39].	
**Data collection instruments and technologies** - Description of instruments (e.g., interview guides, questionnaires) and devices (e.g., audio recorders) used for data collection; if/how the instrument(s) changed over the course of the study<br><br>*We guided with a semi-structured interview guideline through which the participants could access their experiences and reflect upon the underlying antecedents in a layered introspective journey [35]. Before the first interview, we pre-tested the guideline with a health economist, a business information systems researcher, a nurse, and a physician. We evaluated the guide after each interview and iteratively developed it further. Any misconceptions were cleared up and integrated in four iterations of the interview guide, which led to more focused questions about the research topic and better information for the participants during the interview. Thereby, we followed the recommendations of Corbin and Strauss [36].*	p. 5-6
**Units of study** - Number and relevant characteristics of participants, documents, or events included in the study; level of participation (could be reported in results)<br><br>*In total, we interviewed 26 participants and therefore include 26 transcripts into our study.*	p. 9
**Data processing** - Methods for processing data prior to and during analysis, including transcription, data entry, data management and security, verification of data integrity, data coding, and anonymization/de-identification of excerpts<br><br>*For data transcription, we used the software f4analyse and for coding, we used MaxQDA. Data is saved on a protected server and only accessible by the mentioned four authors upon request and in excerpts to guarantee anonymity of participants.*	p. 7
**Data analysis** - Process by which inferences, themes, etc., were identified and developed, including the researchers involved in data analysis; usually references a specific paradigm or approach; rationale**<br><br>*See section "data collection methods" above, as this study follows an iterative data collection and analysis approach.*	p. 5-6

**Techniques to enhance trustworthiness** - Techniques to enhance trustworthiness and credibility of data analysis (e.g., member checking, audit trail, triangulation); rationale**<br><br>*For Trustworthiness:*<br>*Starting from the interview guideline development, we evaluated the guide after each interview and iteratively developed it further to enhance the adherence to the research aim. This also included refinements whenever participants had questions or were discontent/ alerted during the interview, e.g., by clarifying our position towards their management or this studies' implications. With the consent form and our guarantee, that the interviews were pseudonymized, we enhanced trustworthiness.*<br><br>*For Credibility:*<br>*Within our research team, we were aware of potential biases in the data (e.g., social desirability bias), which we tried to mitigate by following recommendations and strategies of Bergen and Labonté [37]. Further, we were aware of potential biases within the team (e.g., confirmation bias of own experiences) and challenged the coding results collectively. Any discrepancies in the allocations of codes, categories, concepts, or relationships between these, were discussed to find agreements.*	p. 5-6

Results/findings

**Synthesis and interpretation** - Main findings (e.g., interpretations, inferences, and themes); might include development of a theory or model, or integration with prior research or theory<br><br>*From the interview transcripts, we derive 506 open codes, which we cluster into three Direct Causes (Organizational Prerequisites, Human Fac-tor, System), and four Influencing Factors (Regulations, Sector Funding, Role of Software Providers, Role of Ownership and Management). While Influencing Factors constitute higher-level influences, they do not directly impact nurses' and physicians' behavior but rather depict the defaults that lead to conditions for Direct Causes of workarounds.*	p. 7
**Links to empirical data** - Evidence (e.g., quotes, field notes, text excerpts, photographs) to substantiate analytic findings<br><br>*Per concept, we provide exemplary quotes to substantiate our findings. Further, we incorporate exemplary workarounds to describe the behavioral status quo.*	p. 10-16

Discussion

**Integration with prior work, implications, transferability, and contribution(s) to the field -** Short summary of main findings; explanation of how findings and conclusions connect to, support, elaborate on, or challenge conclusions of earlier scholarship; discussion of scope of application/generalizability; identification of unique contribution(s) to scholarship in a discipline or field<br><br>*Our research contributes to the theoretical application of the Theory of Planned Behavior (TPB) within the context of Health Information Systems (HIS) workarounds. By identifying specific Direct Causes and Influencing Factors that shape attitudes, norms, and perceived control over workarounds, the study enhances the TPB's explanatory and predictive power, particularly in healthcare settings. This research aligns with and expands on Soffer et al. (2023) by*	p. 21-22

distinguishing between goal-driven behaviors in patient care versus other contexts, emphasizing the ethical and organizational priorities unique to healthcare.<br><br>*The study also broadens the literature on workarounds and human error by incorporating both individual and contextual factors. While the findings are specific to healthcare, the framework has potential applicability in other sectors with similar IT dependencies and regulatory constraints. Unique contributions include a detailed integration of the TPB with the Theory of Workarounds and practical recommendations for stakeholders to address HIS-related issues, including a call for policy changes to promote competition and innovation among HIS providers.*	
**Limitations** - Trustworthiness and limitations of findings<br><br>*The study utilized qualitative exploratory interviews, which are not designed to yield generalizable results. However, these interviews provided a nuanced understanding of the complex motivations behind nurses' and physicians' behaviors, particularly in relation to workaround behaviors. The study relied on self-reported data from participants, which may introduce biases, as participants might not fully disclose all relevant behaviors or may present themselves more favorably. To mitigate this, efforts were made to build rapport and ensure the confidentiality of responses, distancing the researchers from management [66]. The study acknowledges that its findings could have been enhanced by triangulating data with observational methods or system feature assessments and tracking data. This would have provided quantifiable evidence of HIS usage patterns and cross-referenced the qualitative findings [67, 68]. Some interviews were conducted during off-peak times, leading to shorter sessions due to participants' emergent work commitments. Although these interviews were consistent with earlier findings, the reduced time may have limited the depth of information collected. Relationships between identified factors and constructs like Attitude Towards Execution of Workaround, Subjective Norm, and Perceived Behavioral Control were not quantitatively tested or validated. This presents opportunities for future research, although existing literature was used to support assumptions in this study. The study focused on identifying antecedents of workaround behaviors without exploring the cause-effect relationships between these antecedents and specific workarounds employed by medical personnel.*	p. 22-23

Other

**Conflicts of interest** - Potential sources of influence or perceived influence on study conduct and conclusions; how these were managed<br><br>*None declared*	p. 24
**Funding** - Sources of funding and other support; role of funders in data collection, interpretation, and reporting<br><br>*None declared*	n/a
